# Epithelium-on versus epithelium-off corneal collagen crosslinking for keratoconus: a systematic review and meta-analysis

**DOI:** 10.1007/s00417-023-06287-8

**Published:** 2023-11-08

**Authors:** Grace A. Borchert, Himal Kandel, Stephanie L. Watson

**Affiliations:** The University of Sydney, Save Sight Institute, Sydney, New South Wales Australia

**Keywords:** Crosslinking, Epithelium, Keratoconus, Transepithelial

## Abstract

**Purpose:**

Corneal collagen crosslinking (CXL) is the primary treatment for progressive keratoconus which has a significant impact on vision and quality of life. Our study aimed to compare the efficacy and safety of epithelium-on versus epithelium-off CXL to treat keratoconus.

**Methods:**

We searched PubMed, Medline, Embase, Web of Science, and Scopus databases. We included studies that compared standard epithelium-off with epithelium-on CXL. The primary outcome measures were changes in corrected distance visual acuity (CDVA) and maximum keratometry (Kmax), and the secondary outcomes were uncorrected distance visual acuity (UDVA), central corneal thickness (CCT), and adverse events. A meta-analysis was performed on the primary and secondary outcomes based on the weighted mean differences between baseline to 12-month follow-up.

**Results:**

The search retrieved 887 publications with 27 included in the systematic review. A total of 1622 eyes (1399 patients; age 25.51 ± 4.02 years) were included in comparisons of epithelium-off to epithelium-on CXL in keratoconus. Epithelium-off CXL treated 800 eyes and epithelium-on CXL for 822 eyes. At 12-month follow-up, CDVA and Kmax showed no significant difference between the epithelium-off and epithelium-on CXL. The secondary outcomes showed that UDVA was better in epithelium-off CXL (− 0.11*D*, 95% CI − 0.12, − 0.1; *p* < 0.001) and there was more thinning in CCT in epithelium-off CXL (− 3.23 μm, 95% CI − 4.64, − 1.81; *p* <0.001).

**Conclusion:**

Epithelium-off and epithelium-on CXL were both effective to treat progressive keratoconus. Further research is needed to compare the long-term outcomes and safety of both CXL protocols for adaptation into clinical practice.

**Supplementary Information:**

The online version contains supplementary material available at 10.1007/s00417-023-06287-8.


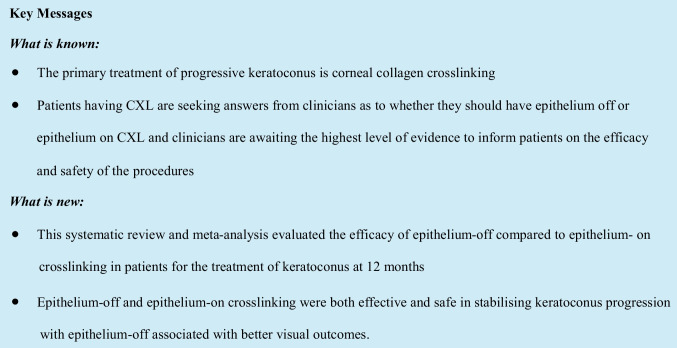


## Introduction

Keratoconus derives from the Greek words ‘kéras,’ meaning cornea and ‘cõnus’ meaning cone, which together gives ‘cone-shaped’ cornea [1]. It is a bilateral asymmetric condition causing progressive corneal thinning and steepening and leading to irregular astigmatism and decreased visual acuity [2]. Epidemiological studies estimate keratoconus prevalence to be between 0.2 and 4790 people per 100,000 with an incidence from 1.5 to 25 per 100,000 people each year [3–6]. Keratoconus and crosslinking have a significant impact on quality of life [7, 8].

The primary treatment of progressive keratoconus is corneal collagen crosslinking (CXL) [9–11]. The CXL procedure aims to stabilize and prevent progression by enhancing the mechanical stability of the cornea [12]. In CXL, riboflavin acts as a photosensitizer to ultraviolet radiation to strengthen the collagen bonds in the cornea [13]. Typically, the CXL procedure involves removal of the epithelium (epithelium-off CXL) to enhance riboflavin penetration although this has been adapted over time [14, 15]. Crosslinking can also be performed with an intact epithelium (epithelium-on CXL) in an effort to decrease the risks associated with removal of the epithelium including microbial keratitis, delayed re-epithelisation, discomfort, haze, corneal edema, and thinning of the cornea [16–18]. However, the corneal epithelium acts as a barrier for riboflavin diffusion which may hinder the CXL photochemical reaction and thus may compromise efficacy of the procedure [19].

A Cochrane meta-analysis of 6 studies reported that there was no evidence of a difference in maximum keratometry (Kmax) and corrected distance visual acuity (CDVA) at 12-month follow-up [20]. There have been a further three meta-analyses which also showed no convincing suggestion for the superiority of epithelium-off or epithelium-on CXL [21–23]. To our knowledge, our study has the largest number of included studies compared to previous studies. Patients having CXL are seeking answers from clinicians as to whether they should have epithelium-off or epithelium-on CXL and clinicians are awaiting the highest level of evidence to inform patients on the efficacy and safety of the procedures. We performed this study to add to the evidence base to answer these questions.

The aim of this study was using available evidence to assess the efficacy and safety of epithelium-off with epithelium-on CXL.

## Method

A systematic review and meta-analysis of studies comparing epithelium-off to epithelium-on CXL in patients with keratoconus was performed with reference to the Cochrane and Centre for Reviews and Dissemination. The review follows the Preferred Reporting Items for Systematic Reviews and Meta-Analyses statement (PRISMA) guidelines [24].

### Search strategy

**Fig. 1:**
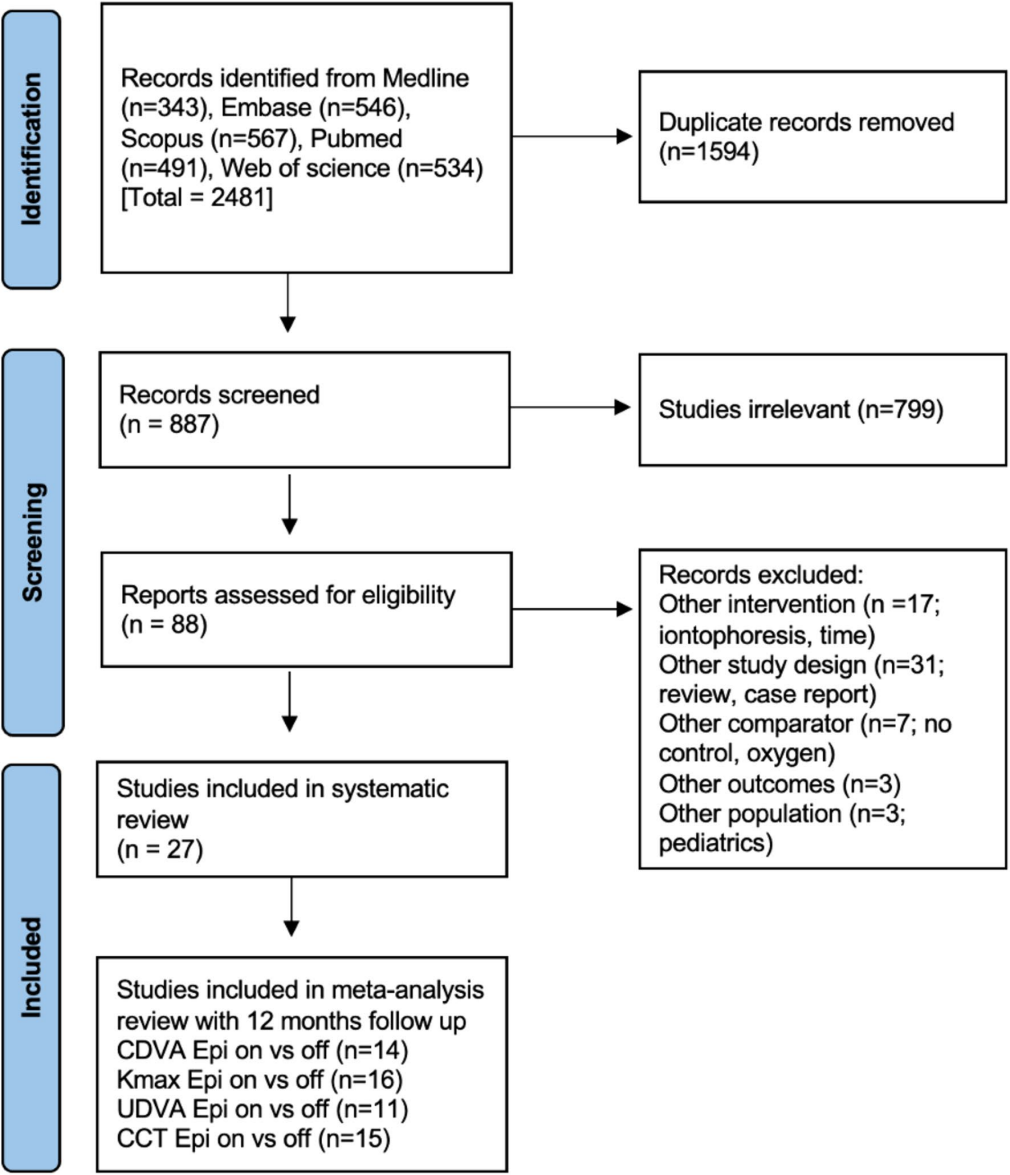
Preferred Reporting Items for Systematic Reviews and Meta-analyses (PRISMA) flowchart showing the search process to identify relevant articles for inclusion

A systematic literature search was conducted in Medline, Embase, PubMed, Scopus, and Web of Science databases to May 2022. The search strategy is outlined in emental Digital Content 1. The main search concepts were the population (patients with keratoconus), treatment (crosslinking), and specific surgical technique (epithelium off and on). The search was restricted to studies within the last 10 years and in English. A search in the grey literature was performed in OpenGrey. The reference lists were hand-searched to identify additional studies. Fig. 1 is a flowchart based on PRISMA for the study.

### Study selection, screening, and data extraction

We included studies that compared outcomes on the efficacy and safety of epithelium-off to epithelium-on CXL in patients with keratoconus. Patients were included in meta-analysis if the CDVA and Kmax at 12 months were compared. The population was restricted to adult patients and non-human studies were excluded.

The publications were screened by two authors (GB and HK) and discrepancies resolved by consensus (GB, HK, and SW). All retrieved articles were exported to Covidence to remove duplicates, screen titles, and abstracts and for a full-text review. The data of interest included: study data (author, year of publication), country in which the study was country, patient demographics, study characteristics, CDVA, uncorrected distance visual acuity (UDVA), corneal parameters (K2, Kmax), central corneal thickness (CCT), and adverse events.

### Risk of bias

The risk of bias was determined for each study using the National Health and Medical Research Council guidelines [25]. The risk of bias for the randomized control trials (RCTs) was evaluated by the Cochrane RoB 2.0 tool and the studies that were non-randomized prospective studies were assessed by ROBINS-I [26–28]. This risk of bias assessment was conducted by two reviewers (GB and HK) independently and disagreements were resolved through discussion with the third reviewer (SW). The risk of bias figures were generated using the Risk-of-bias Visualisation (Robvis) tool which uses an R package and Shiny web app to visualize the risk of bias assessment (Supplemental Digital Content 4 and Supplemental Digital Content 5) [29].

### Outcome measures

The primary outcomes were the mean difference in CDVA and Kmax from baseline to 12-month follow-up. This timeframe was assessed since 12 months was the most consistently reported in the included studies. Secondary outcomes included uncorrected distance visual acuity (UDVA), central corneal thickness (CCT), and adverse events.

### Statistical analysis

A meta-analysis was performed using a random-effects model to determine the weighted effect estimation. Possible heterogeneity between studies was measured with the *I*^2^ statistic. Statistical analysis and meta-analysis were performed using Review Manager (open-source desktop v5.4) developed by Cochrane Collaborations. A *p*-value less than 0.05 was considered statistically significant.

## Results

### Study selection

The search yielded 887 publications after duplicates were removed. The search results were screened with 88 full texts assessed for eligibility, leaving 27 included studies for the systematic review (Fig. 1). A meta-analysis was performed on various subsets based on matching the variables and follow-up time periods. Records were excluded for several reasons including wrong intervention, study design, comparator, outcomes, and population.

The patient demographics for each study are shown in Table 1. The included studies contained a total of 1399 patients with keratoconus with 1622 eyes either treated with epithelium-off or epithelium-on CXL. The overall mean age was 25.5 (SD 4.02) years with follow up ranging from 1 week to 5 years. There were 49.3% of all eyes had epithelium-off CXL.

**Table 1:** Characteristics of included studies for the systematic review. Key Epi-on = epithelium-on CXL, Epi-off = epithelium-off CXL

Included study	Total patients (*n*)	Eyes Epi-on (*n*)	Eyes Epi-off (*n*)	Age Epi-on (years)	Age Epi-off (years)	Sex female (*n*, %)	Sex male (*n*, %)	Country
Abdelkader 2021 [30]	69	35	34	24.9	24.9	20, 29%	49, 71%	Spain
Abdel-Radi 2020 [31]	44	33	41	25.15	23.3	18, 41%	26, 59%	Egypt
Akbar 2017 [32]	64	32	32	24.47	24.81	18, 28%	46, 72%	Taiwan
AlFayez 2015 [33]	70	34	36	24.8	24.1	39, 56%	31, 44%	Saudi Arabia
AlZubi 2019 [34]	80	40	40	23.55	22.89	20, 25%	60, 75%	Jordan
Arance-GilÁ 2021 [17]	46	33	31	25.33	19.9			Spain
Badawi 2021 [35]	36	29	41	22.22	23.44	14, 39%	22, 61%	Egypt
Çerman 2015 [36]	39	30	30	22.8	23.7			Turkey
Cifariello 2018 [37]	32	20	20	31	24	11, 34%	29, 91%	Italy
Godefrooij 2017 [38]	61	35	26	26.9	25.9	14, 23%	47, 77%	Netherlands
Huang 2020 [39]	70	34	26	19.6	21.5	11, 16%	28,4 0%	China
Kocak 2014 [40]	36	17	19	27.35	27.16	19, 53%	17, 47%	Turkey
Madeira 2019 [41]	24	16	10	18.69	21.9	3, 13%	21, 88%	Portugal
Nawaz 2015 [42]	40	20	20	22.35	23.95	8, 20%	32, 80%	India
Nicula 2020 [43]	169	76	93	29	26.5	76, 45%	93, 55%	Romania
Niyazmand 2021 [44]	61	34	27	25.8	25.8	29, 48%	32, 52%	Australia
Ouyang 2021 [45]	40	30	30	21.1	21.1			China
Rossi 2015 [46]	20	10	10	28.3	30.4	9, 45%	11, 55%	Italy
Rossi 2018 [47]	20	10	10	27.2	30.4	10, 50%	10, 50%	Italy
Rush 2017 [48]	131	75	56	29.8	31.5	43, 33%	88, 67%	USA
Salah 2019 [49]	20	20	20	32	32	11, 55%	9, 45%	Egypt
Soeters 2015 [50]	61	35	26	24	24	14, 23%	47, 77%	Netherlands
Spadea 2015 [51]	50	25	25	31.5	29.2	27, 54%	29, 58%	Italy
Spadea 2018 [52]	36	19	17	37	31.08	19, 53%	17, 47%	Italy
Stojanovic 2014 [53]	20	20	20	29.5	29.5	3, 15%	17, 85%	Norway
Yuksel 2015 [54]	39	39	39	20.1	19.8	11, 28%	28, 72%	Turkey
Yuksel 2020 [55]	21	21	21	20.3	20.3	9, 43%	12, 57%	Turkey
Total	1399	822	800	25.73	25.09	477	841	
Proportion/mean		50.7%	49.3%	25.73	25.3	36.2%	63.8%	

### Primary outcomes

**Fig. 2:**
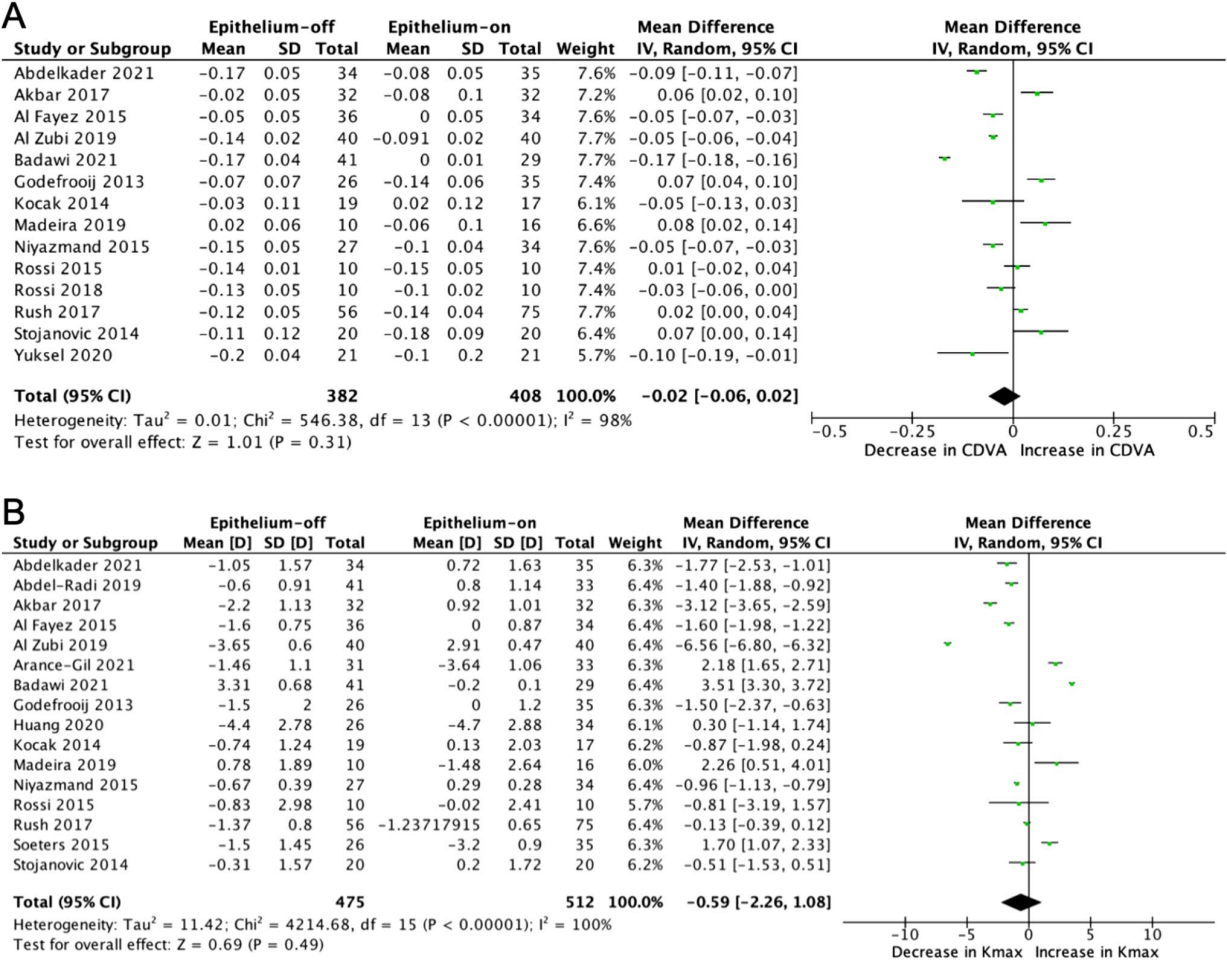
**Forest plot of primary outcomes.** Mean difference **A** CDVA (logMAR) and **B** Kmax (*D*) for epithelium-off vs. epithelium-on corneal CXL. Corrected for the included studies’ respective baseline to 12 months follow up. Key CI = confidence interval, SD = standard deviation

The corrected distance visual acuity (CDVA) improved from baseline to 12-month follow-up in both epithelium-off and epithelium-on CXL (Fig. 2A). The adjusted mean difference of CDVA between epithelium-off and epithelium-on CXL at 12-month follow-up was − 0.02 logMAR (95% CI − 0.06, 0.02; *p* = 0.31). The Kmax decreased in both epithelium-off and epithelium-on CXL from baseline to 12-month follow-up (Fig. 2B). However, the Kmax mean difference was not significant between the epithelium-off and epithelium-on CXL at 12-month follow-up which was − 0.59*D* (95% CI − 2.26, 1.08; *p* = 0.49). Heterogeneity between studies was high (*I*^2^ = 100%; *p* <0.01).

### Secondary outcomes

**Fig. 3:**
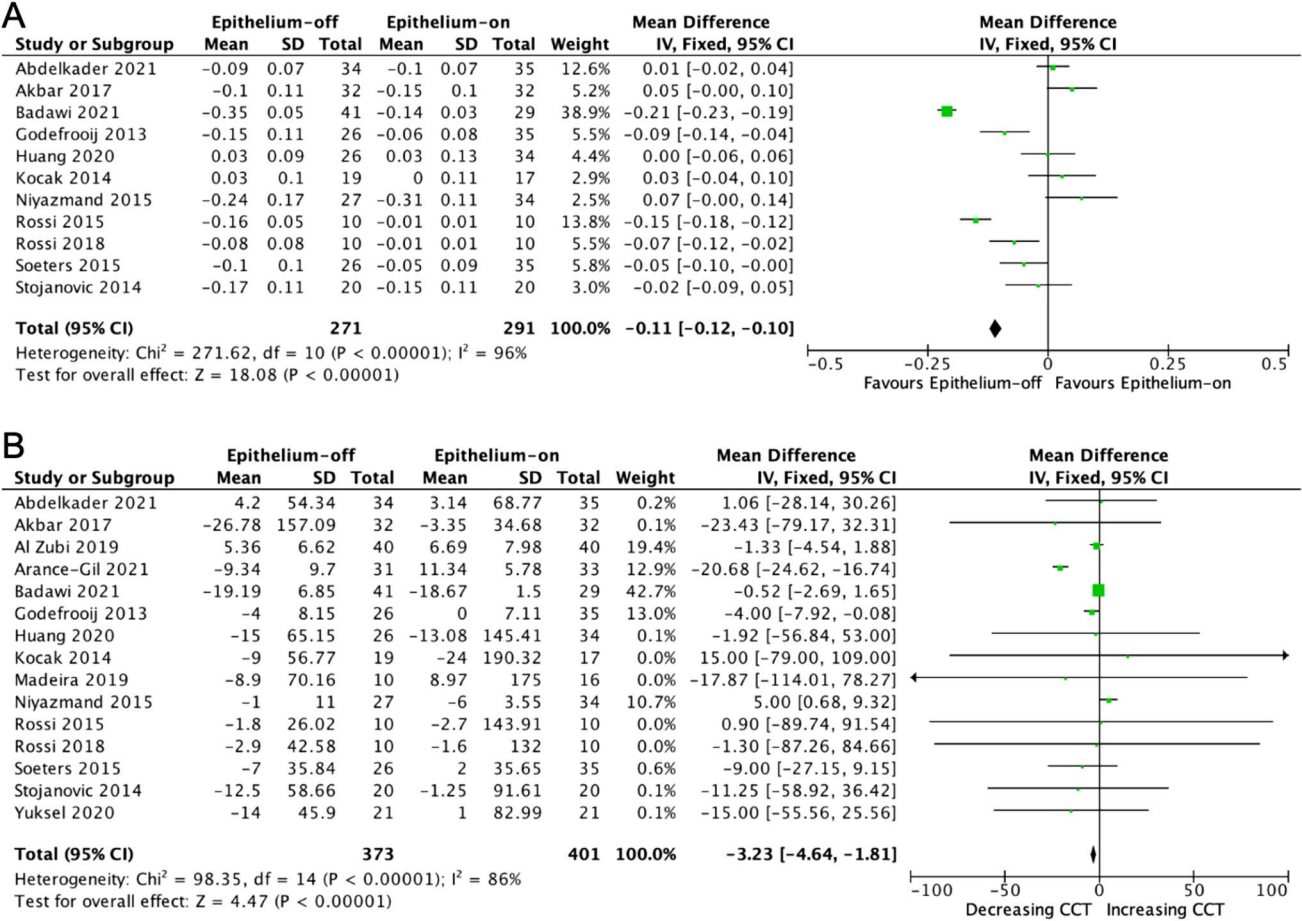
**Forest plot of secondary outcomes.** Mean difference: **A** uncorrected distance visual acuity (UDVA) and **B** central corneal thickness (CCT) for epithelium-off and epithelium-on corneal CXL

The uncorrected distance visual acuity (UDVA) significantly improved in the epithelium-off compared to the epithelium-on group CXL (Fig. 3A). The UDVA adjusted mean difference was − 0.11 logMAR (95% CI − 0.12, − 0.1; *p* <0.001). Heterogeneity between studies was high (*I*^2^ = 96%; *p* <0.01). The reduction in mean central corneal thickness (CCT) was significantly higher in the epithelium-off group compared to the epithelium-on CXL group (Fig. 3B). The adjusted mean difference in CCT between the CXL protocols was − 3.23μm (95% CI − 4.64, − 1.81; *p* <0.001). Heterogeneity between studies was high (*I*^2^ = 86%; *p* <0.01).

Adverse events were not reported in 20 studies out of the total 27 studies included in the systematic review. There were 7 studies that described complications or adverse events, and each varied in reporting, follow up, and management (Table 2). Overall, there were more adverse events described in the epithelium-off CXL group compared to the epithelium-on CXL group. In the epithelium-on CXL group, there was no adverse events reported except in Salah 2019 [49] and Badawi 2021 [35] with mild corneal haze.

**Table 2:** Adverse events having epithelium-off compared to epithelial-on crosslinking

Adverse event	Study	Epithelium-off *n*, percentage (%)	Timeframe	Epithelium-on *n*, percentage (%)	Timeframe
Corneal haze, *n* (%total)	Abdelkader 2021 Salah 2019 Kocak 2014 Soeters 2015 Al Zubi 2019 Badawi 2021	3 (8.8) 9 (45) 14 (74) 1 (4) 4 (10) 27 (66)	PO1 d NS NS 1 m 3–4 m 1–3 m	0 4 [2] 0 0 0 5 [18]	PO 1 d NS NS 1 m 3–4 m 1–3 m
Scarring, *n* (% total)	Soeters 2015	1 (4)	1 w	0	1 w
Sterile infiltrates, *n* (%total)	Soeters 2015, Abdelkader 2021	1 (4) 1 (3)	1 w NS	0 0	1 w NS
Epithelial defect	Abdelkader 2021	1 (3)	3, 6 m	0	3, 6 m
Herpes keratitis	Soeters 2015	1 (4)	1 w	0	1 w
Pain	Rossi 2018	Reported but NS	NS	0	NS

*n* number, *NS* not specified, *PO* postoperative, *d* day(s), *w* week(s), *m* month(s)

Adverse events included corneal haze, scarring, sterile infiltrates, epithelial defect, and herpes keratitis. Corneal haze was the most common of the adverse events reported in the epithelium-off CXL group. Badawi et al. [35] measured corneal haze both subjectively and objectively at 3-, 6-, and 12-month follow-up. It was found that the epithelium-on CXL group had a lower corneal haze incidence, with a better and quicker recovery. It was observed that there was one case of an epithelial defect which resolved and completely closed by the third postoperative day. There was one case of delayed epithelial healing which resulted at 3 months in a stromal haze and at 6 months deep stroma haze.

## Discussion

This systematic review and meta-analysis compared the efficacy and safety of epithelium-off versus epithelium-on CXL in the treatment of keratoconus. Both techniques were effective at improving the visual acuity and keratometry outcomes. However, the primary outcomes showed no significant difference between epithelium-off and epithelium-on CXL after 12 months based on CDVA or Kmax. The UDVA improved more in the epithelium-off CXL (mean difference in gain of − 0.11D 95% CI − 0.12, − 0.1; *p* < 0.001) and CCT reduction was higher in the epithelium-off CXL (mean difference between the protocols − 3.23 μm, 95% CI − 4.64, − 1.81; *p* <0.001). Adverse events were reported in an average of 26.7% in epithelium-off and 3.3% in epithelium-on CXL (*p* < 0.05). The study findings support both CXL techniques as safe and effective for patients with keratoconus at 12 months.

Our findings comparing epithelium-off and epithelium-on CXL are representative of and consistent with previously reported studies. A Cochrane systematic review and meta-analysis showed no difference in Kmax and demonstrated epithelium-off had more adverse events [20]. A meta-analysis by Zhang et al. demonstrated no significant difference in CDVA or Kmax but also showed a significant difference in CCT (mean difference in change 4.53; 95% CI 0.42, 8.64; *p* = 0.03) to a similar effect size evaluated in our study [56]. Meanwhile, D’Oria et al. demonstrated a CDVA mean difference between epithelium-off and epithelium-on CXL of 0.07logMAR (CI 0.04, 0.1; *p* < 0.001). Epithelium-on CXL was shown to have significant less risk of delay in epithelial healing (*p* = 0.035) and persistent stromal haze (*p* = 0.026). Although no significant differences in UDVA, Kmax, and CCT [57]. Most studies did not include adverse events although those that did reported a higher incidence in epithelium-off CXL compared to epithelium-on CXL. The lack of reported adverse events highlights an area for further research.

While the method of epithelium-off CXL followed the Dresden protocol, there was variation in the epithelium-on CXL protocol methodology. For example, there was variation in the methods used to increase epithelial permeability of riboflavin in the epithelium-on CXL group for example Al Fayez et al. [33] used benzalkonium chloride, Soeteres et al. [50] used Ricrolin TE solution, and Rush et al. [48] used benzalkonium chloride and hydroxypropyl methylcellulose. There was considerable heterogeneity within the primary and secondary outcomes in comparing epithelium-on and epithelium-off CXL. Adverse events were only reported in 7 out of 19 included studies. This suggests that there is a lack of reporting standards in the literature. In the studies describing adverse events, there was discrepancies in the follow-up and treatment.

The rationale for epithelial removal during CXL has been to facilitate the diffusion of riboflavin into the corneal stroma. It has been suggested that the corneal biomechanical rigidity in rabbits’ eyes following epithelium-on CXL is one-fifth of that following epithelium-off CXL [58]. Leaving the epithelium intact may decrease the risks of microbial keratitis, delayed re-epithelialisation, discomfort, haze, corneal edema, glare, and thinning of the cornea [16, 17]. In studies evaluating epithelium-on CXL, minimal haze has been reported. In standard epithelium-off CXL haze occurs frequently as the epithelium is removed triggering keratocyte apoptosis and proliferation of myofibroblasts [37]. There have been concerns as to the effectiveness of epithelium-on CXL as the epithelium acts as a barrier to diffusion of riboflavin. Methods including iontophoresis or ways to enhance the chemical permeability, for example, using benzalkonium chloride have been trialled to overcome the barriers to riboflavin diffusion with an intact epithelium [59].

The strength of our study was that, to our knowledge, it is the most comprehensive systematic review and meta-analysis that compares epithelium-off to epithelium-on CXL in keratoconus. The natural course of keratoconus is life long and this systematic review and meta-analysis focused on 12-month follow-up. It has been demonstrated that outcomes can continue to change over time in CXL such that future studies should include medium to long-term outcomes. Recently, the five-year CXL outcomes were published from the Save Sight Keratoconus Registry which showed CXL was effective in stabilizing keratoconus up to 5 years in most patients [60]. This systematic review and meta-analysis were limited by the quality of the studies included, heterogeneity, biases, small sample size, and short-term follow-up. Our study was also limited by the heterogeneity of the included studies. There have been very few studies that have investigated the adverse events of epithelium-off and epithelium-on CXL with limited data reported on the adverse event described, along with variable follow-up times and different approaches in management. The complete picture of the safety and efficacy of epithelium-off vs. epithelium-on CXL is still emerging. Our findings may provide a foundation for future studies of epithelium off vs. on CXL.

## Conclusion

In summary, both epithelium-off and epithelium-on CXL groups were effective in improving visual and keratometry outcomes. The primary purpose of CXL is to stop or slow the progression and stabilize keratoconus which both techniques effectively achieved. There was no difference at 12-month follow-up in terms of CDVA and Kmax. Further research is needed to compare the long-term safety and efficacy between the two techniques.

### Supplementary information

ESM 1 **Supplemental Digital Content 1.** Search Strategy (DOCX 17 kb)

Fig. 4 **Supplemental Digital Content 2.** ROBINS-I Risk of Bias (PNG 173 kb)

High resolution image (TIFF 924 kb)

Fig. 5 **Supplemental Digital Content 3.** RoB2 (PNG 865 kb)

High resolution image (TIFF 186 kb)
