# Supplementary material for: Epithelium-on versus epithelium-off corneal collagen crosslinking for keratoconus: a systematic review and meta-analysis
Source: Graefes Arch Clin Exp Ophthalmol. 2023 Nov 8;262(6):1683–92. doi: 10.1007/s00417-023-06287-8 (PMC11106102; doi:10.1007/s00417-023-06287-8)
Supplement: Supplementary file 1 — Supplemental Digital Content 1. Search Strategy (DOCX 17 kb) [file 417_2023_6287_MOESM1_ESM.docx]

**Supplemental Digital Content 1. Search Strategy**

Search results retrieved until 19/05/2022

**Pubmed**

( "keratoconus" OR "keratoconic" ) AND ( "cross-linking" OR "crosslinking" OR "cross linking" OR "corneal cross-linking" OR "corneal crosslinking" OR "corneal cross linking" OR "corneal collagen cross-linking" OR "corneal collagen crosslinking" OR "corneal collagen cross linking" OR "conventional corneal collagen cross-linking" OR "conventional corneal collagen crosslinking" OR "conventional corneal collagen cross linking" OR "CXL" ) AND ( "epithelium" OR "epithelium on" OR "epithelium off" OR "epithelium-on" OR "epithelium-off" OR "epi-on" OR "epi-off" OR "epithelium removal" OR "transepithelial" )

Restricted to 2013 – 2022

English only

N=465

**Medline**

**Supplemental Table 1. Master keyword search for Medline via OvidSP**

| **#** | **Searches** | **Results** |
| --- | --- | --- |
| **1** | keratoconus/ | 9761 |
| **2** | keratoconus*.mp. | 10562 |
| **3** | 1 or 2 | 10562 |
| **4** | collagen/ | 136269 |
| **5** | collagen*.mp. | 373438 |
| **6** | cross-link*.mp. | 129208 |
| **7** | (collagen cross adj2 link*).mp. | 3489 |
| **8** | collagen crosslink*.mp. | 1885 |
| **9** | (CCL or CXL).tw. | 7845 |
| **10** | 4 or 5 or 6 or 7 or 8 or 9 | 350934 |
| **11** | Epithelium/ | 71696 |
| **12** | Epithelium*.mp. | 219258 |
| **13** | Transepithelial/ | 0 |
| **14** | 11 or 12 or 13 | 219258 |
| **15** | 3 and 10 and 15 | 442 |
| **16** | Limit 15 to yr=”2013-Current” | 291 |
| **17** | Limit 16 to English language | 274 |

N=274

**Embase**

**Supplemental Table 2. Master keyword search for Embase via OvidSP**

| **#** | **Searches** | **Results** |
| --- | --- | --- |
| **1** | keratoconus/ | 10288 |
| **2** | keratoconus*.mp. | 11122 |
| **3** | 1 or 2 | 11122 |
| **4** | collagen/ | 140741 |
| **5** | collagen*.mp. | 388294 |
| **6** | cross-link*.mp. | 134372 |
| **7** | (collagen cross adj2 link*).mp. | 3610 |
| **8** | collagen crosslink*.mp. | 2017 |
| **9** | (CCL or CXL).tw. | 8216 |
| **10** | 4 or 5 or 6 or 7 or 8 or 9 | 511964 |
| **11** | Epithelium/ | 66538 |
| **12** | Epithelium*.mp. | 461775 |
| **13** | Transepithelial/ | 0 |
| **14** | 11 or 12 or 13 | 461775 |
| **15** | 3 and 10 and 15 | 703 |
| **16** | Limit 15 to yr=”2013-Current” | 568 |
| **17** | Limit 16 to English language | 546 |

N=546

**Scopus**

( TITLE-ABS-KEY ( "keratoconus"  OR  "keratoconic" )  AND  TITLE-ABS-KEY ( "cross-linking"  OR  "crosslinking"  OR  "cross linking"  OR  "corneal cross-linking"  OR  "corneal crosslinking"  OR  "corneal cross linking"  OR  "corneal collagen cross-linking"  OR  "corneal collagen crosslinking"  OR  "corneal collagen cross linking"  OR  "conventional corneal collagen cross-linking"  OR  "conventional corneal collagen crosslinking"  OR  "conventional corneal collagen cross linking"  OR  "CXL" )  AND  TITLE-ABS-KEY ( "epithelium"  OR  "epithelium on"  OR  "epithelium off"  OR  "epithelium-on"  OR  "epithelium-off"  OR  "epi-on"  OR  "epi-off"  OR  "epithelium removal"  OR  "transepithelial" ) )  AND  ( LIMIT-TO ( PUBYEAR ,  2022 )  OR  LIMIT-TO ( PUBYEAR ,  2021 )  OR  LIMIT-TO ( PUBYEAR ,  2020 )  OR  LIMIT-TO ( PUBYEAR ,  2019 )  OR  LIMIT-TO ( PUBYEAR ,  2018 )  OR  LIMIT-TO ( PUBYEAR ,  2017 )  OR  LIMIT-TO ( PUBYEAR ,  2016 )  OR  LIMIT-TO ( PUBYEAR ,  2015 )  OR  LIMIT-TO ( PUBYEAR ,  2014 )  OR  LIMIT-TO ( PUBYEAR ,  2013 ) )  AND  ( LIMIT-TO ( LANGUAGE ,  "English" ) )

N=576

**Web of Science**

"keratoconus" OR "keratoconic" (All Fields) AND "cross-linking" OR crosslinking OR "cross linking” OR "corneal cross-linking" OR "corneal crosslinking" OR "corneal cross linking" OR "corneal collagen cross-linking" OR "corneal collagen crosslinking" OR "corneal collagen cross linking" OR “conventional corneal collagen cross-linking” OR “conventional corneal collagen crosslinking” OR “conventional corneal collagen cross-linking” (All Fields) AND "epithelium" OR "epithelium on" OR "epithelium off" OR "epithelium-on" OR "epithelium-off" OR "epi-on" OR "epi-off" OR "epithelium removal" OR "transepithelial" (All Fields)

Restricted to 2013 to 2022

Restricted to English

N=695
