# Supplementary figures and images for: Epithelium-on versus epithelium-off corneal collagen crosslinking for keratoconus: a systematic review and meta-analysis
Source: Graefes Arch Clin Exp Ophthalmol. 2023 Nov 8;262(6):1683–92. doi: 10.1007/s00417-023-06287-8 (PMC11106102; doi:10.1007/s00417-023-06287-8)

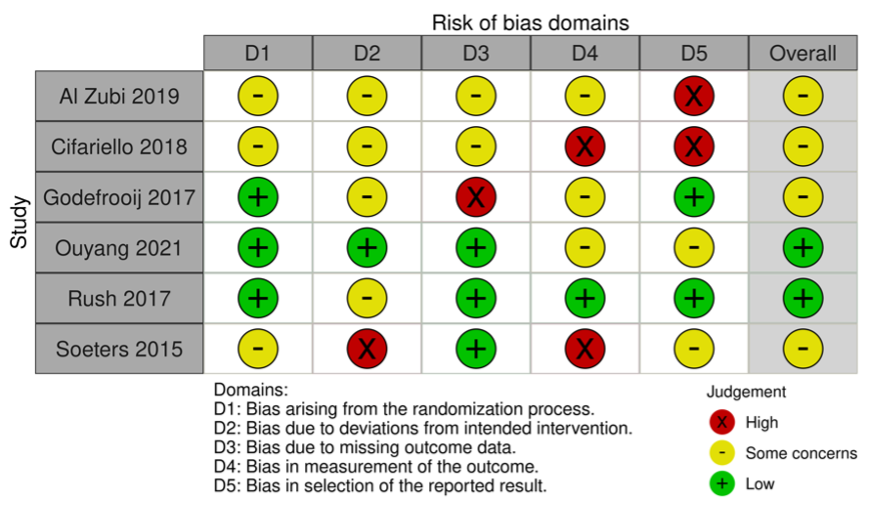

Supplement: Supplementary file 2 — Supplemental Digital Content 2. ROBINS-I Risk of Bias (PNG 173 kb) [file 417_2023_6287_Fig4_ESM.png]

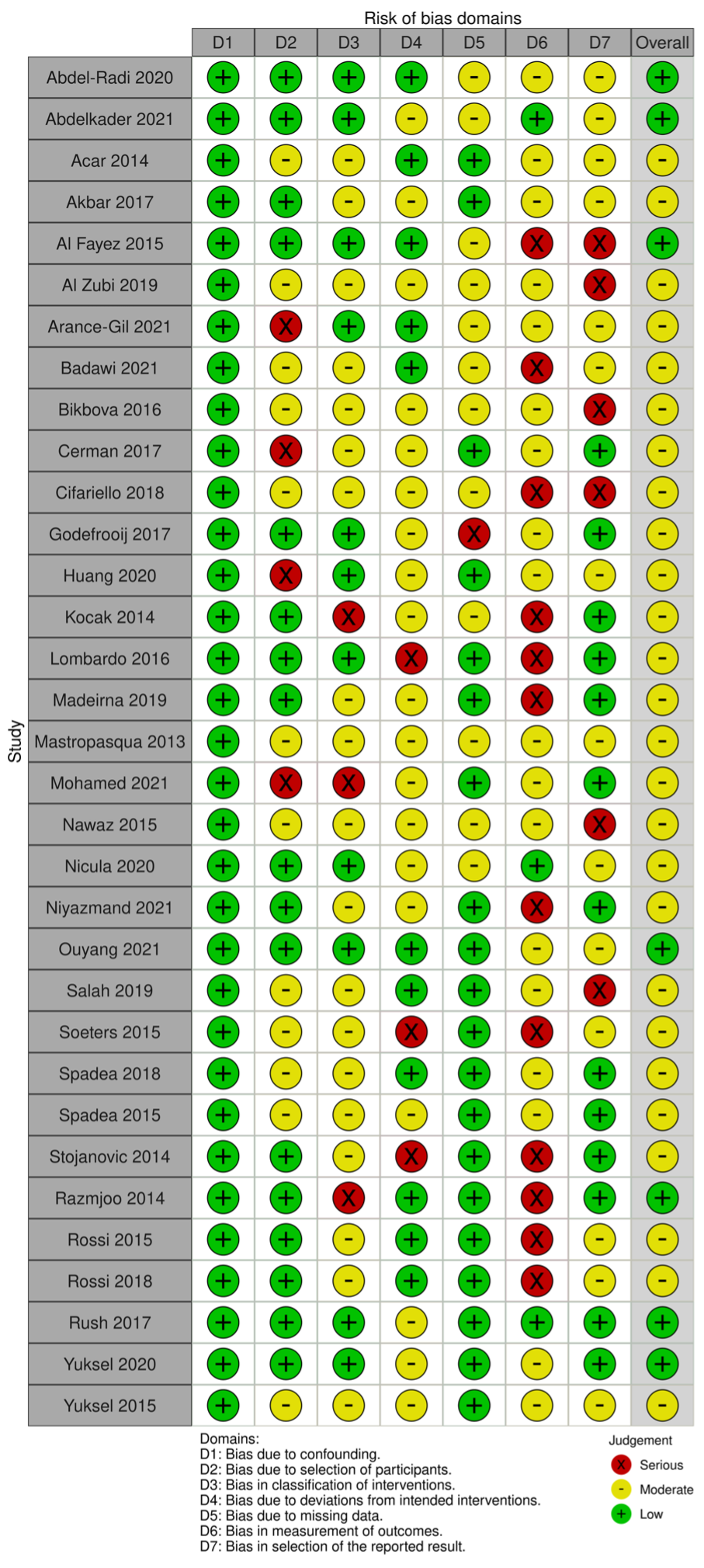

Supplement: Supplementary file 3 — High resolution image (TIFF 924 kb) [file 417_2023_6287_MOESM2_ESM.tiff]

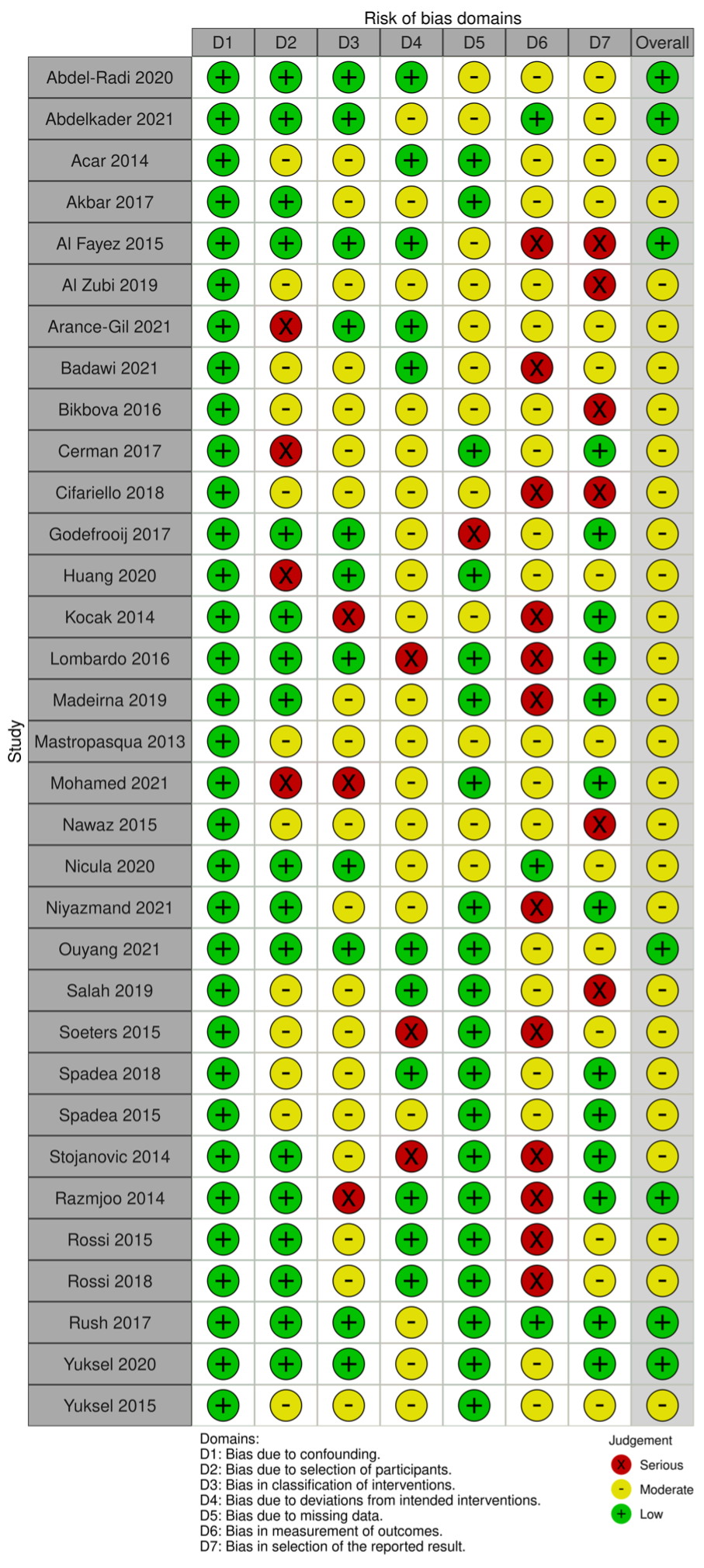

Supplement: Supplementary file 4 — Supplemental Digital Content 3. RoB2 (PNG 865 kb) [file 417_2023_6287_Fig5_ESM.png]

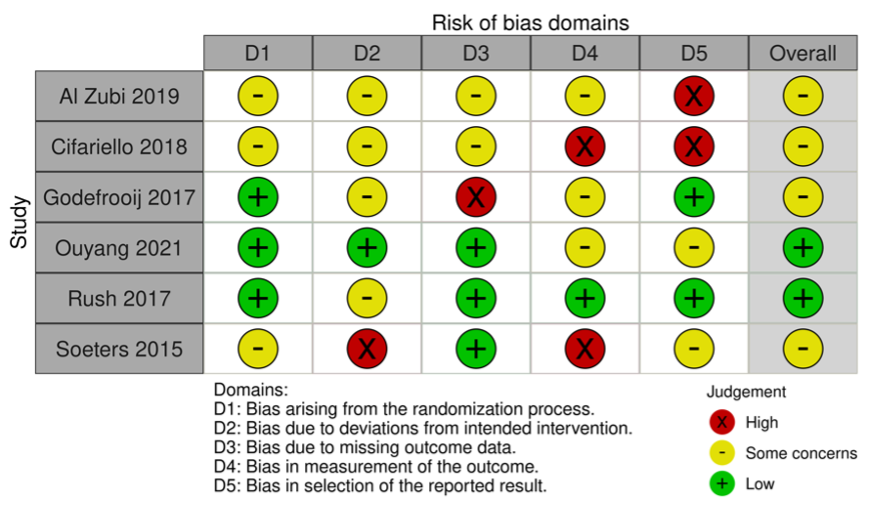

Supplement: Supplementary file 5 — High resolution image (TIFF 186 kb) [file 417_2023_6287_MOESM3_ESM.tiff]
